# The low energy signaling network

**DOI:** 10.3389/fpls.2014.00353

**Published:** 2014-07-17

**Authors:** Filipa Tomé, Thomas Nägele, Mattia Adamo, Abhroop Garg, Carles Marco-llorca, Ella Nukarinen, Lorenzo Pedrotti, Alessia Peviani, Andrea Simeunovic, Anna Tatkiewicz, Monika Tomar, Magdalena Gamm

**Affiliations:** ^1^Bayer CropScience NV, Innovation CenterGhent, Belgium; ^2^Department of Ecogenomics and Systems Biology, University of ViennaVienna, Austria; ^3^Instituto Gulbenkian de CiênciaOeiras, Portugal; ^4^Zentrum für Molekularbiologie der Pflanzen, Eberhard Karls Universität TübingenTübingen, Germany; ^5^Julius-von-Sachs-Institut, Julius-Maximilians-Universität WürzburgWürzburg, Germany; ^6^Theoretical Biology and Bioinformatics, Department of Biology, Faculty of Science, Utrecht UniversityUtrecht, Netherlands; ^7^Universidad Politécnica de Madrid–Instituto Nacional de Investigación y Tecnología Agraria y Alimentaria, Centro de Biotecnología y Genómica de Plantas, Universidad Politécnica de MadridMadrid, Spain; ^8^Molecular Plant Physiology, Institute of Environmental Biology, Utrecht UniversityUtrecht, Netherlands

**Keywords:** energy signaling, TOR, SnRK1, bZIP, T6P, stress, metabolism

## Abstract

Stress impacts negatively on plant growth and crop productivity, caicultural production worldwide. Throughout their life, plants are often confronted with multiple types of stress that affect overall cellular energy status and activate energy-saving responses. The resulting low energy syndrome (LES) includes transcriptional, translational, and metabolic reprogramming and is essential for stress adaptation. The conserved kinases sucrose-non-fermenting-1-related protein kinase-1 (SnRK1) and target of rapamycin (TOR) play central roles in the regulation of LES in response to stress conditions, affecting cellular processes and leading to growth arrest and metabolic reprogramming. We review the current understanding of how TOR and SnRK1 are involved in regulating the response of plants to low energy conditions. The central role in the regulation of cellular processes, the reprogramming of metabolism, and the phenotypic consequences of these two kinases will be discussed in light of current knowledge and potential future developments.

## INTRODUCTION

**FIGURE 1:**
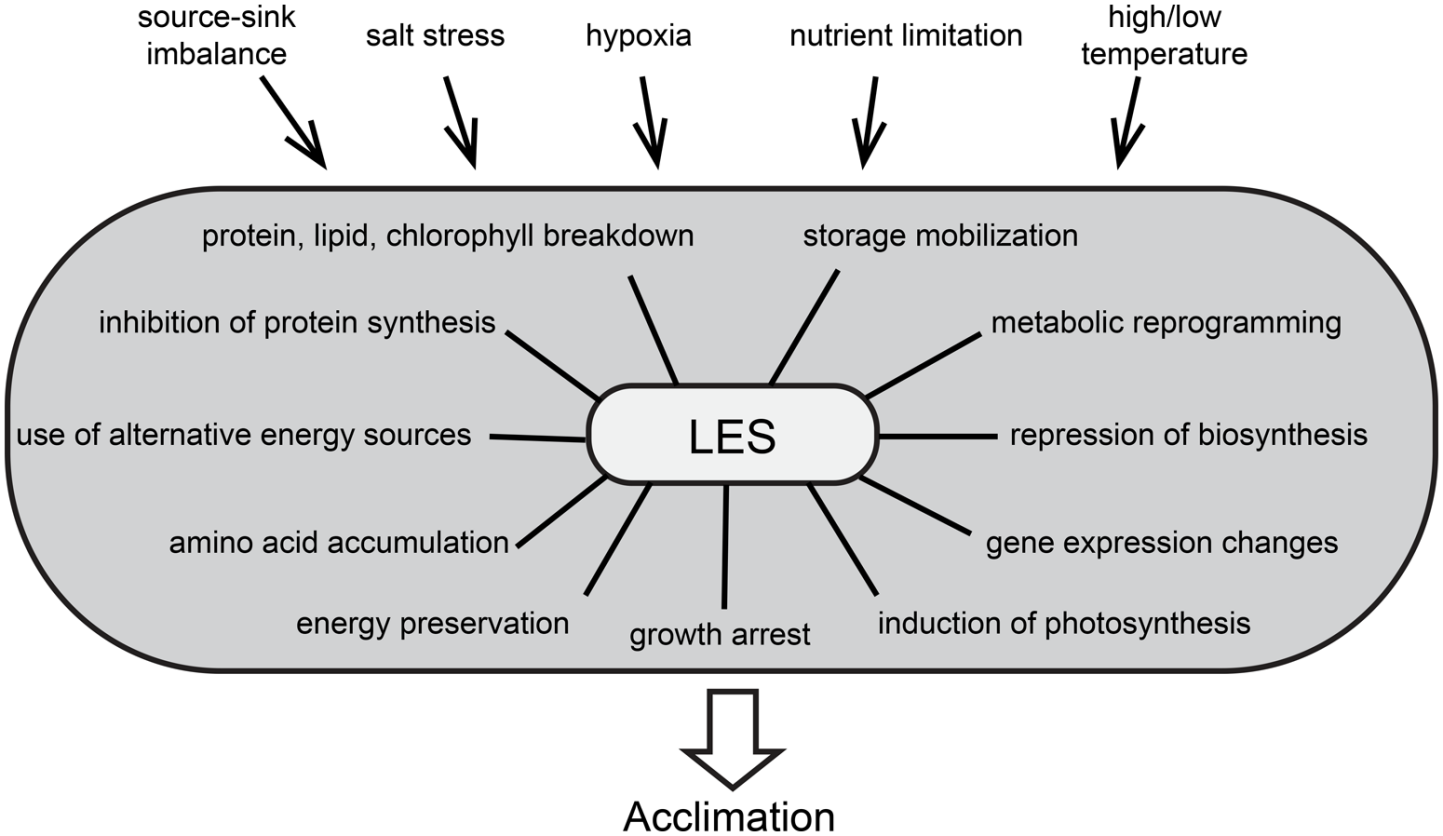
**The low energy syndrome (LES) is a collection of phenotypical consequences of stress condition and drives plant acclimation to environmental changes.** The figure aims at depicting all causes and consequences described so far, but LES can be induced by one or multiple stress conditions and the acclimation process can include only a subset of the depicted outcomes.

Suboptimal growth conditions related to temperature, light, water supply, and soil characteristics are among the most limiting factors for crop yield worldwide. Fruit and seeds constitute about 75% of world crop production (Liu et al., 2013a). They rely on the supply of carbohydrates from photosynthetic source tissues to sustain growth and development (Rolland et al., 2006) and this fine-tuned balance can be severely disturbed at different levels under biotic and abiotic stress conditions (Lemoine et al., 2013). In general, stress conditions cause the alteration of a set of processes and biochemical reactions. These changes can be encompassed by the term low energy syndrome (LES) and play a major role in the adaptation to stress conditions (**Figure 1**). In this review we summarize recent advances in the understanding of LES and the different signaling pathway components from energy deficiency toward adaptation, focusing on *Arabidopsis thaliana* as a plant model.

## THE LOW ENERGY SYNDROME IS PART OF STRESS ADAPTATION

Controlling energy homeostasis is a challenge for all organisms, as they must constantly sense and integrate internal and external signals to optimize growth and development, often under suboptimal conditions (Polge and Thomas, 2007). This is particularly critical for plants as they are sessile organisms, so it is very interesting to understand how they have evolved to overcome these constraints and how they respond differently to stresses when compared to other organisms. Their adaptation and survival depend on their capacity to efficiently manage energy resources in all tissues, and to coordinate energy consumption and preservation (Baena-González et al., 2007; Baena-González, 2010). Stress conditions affect source and sink tissues differently. In plants exposed to stress, sink organs like seeds or tubers often suffer from reduced sugar import and are impaired in biomass production (Pinheiro et al., 2001; Cuellar-Ortiz et al., 2008). Accordingly, stress factors like nutrient limitation, hypoxia, excess of salt, and low or high temperatures were discussed to impair fruit and seed development by interfering with the source-sink balance (Gibon et al., 2002; Geigenberger, 2003; Bailey-Serres et al., 2012; Lemoine et al., 2013; Liu et al., 2013b). Furthermore, stress conditions often affect photosynthesis and respiration in source leaves and this can accentuate the source-sink imbalance. The resulting energy deprivation was suggested to be common to most types of stress and to trigger specific responses (Baena-González and Sheen, 2008), leading to the massive alteration of cellular processes, referred to as LES. This includes growth arrest and metabolic reprogramming, comprising the repression of biosynthetic activities and sugar storage, as well as the induction of catabolic processes, photosynthesis, and sugar remobilization (Paul and Pellny, 2003; Gibon et al., 2006). In addition, the expression of thousands of genes is altered (Usadel et al., 2008). These genes were described to form a network that regulates plant metabolism under stress conditions for the purpose of energy preservation (Avin-Wittenberg et al., 2012). At the same time, metabolic reprogramming favors catabolic processes of molecules other than carbohydrates, resulting in protein, lipid and chlorophyll breakdown (Contento et al., 2004; Thimm et al., 2004). Accordingly, metabolomics data show an increase in amino acids coming from protein degradation (Caldana et al., 2011), which may contribute to sustain levels of TCA cycle intermediates (Araújo et al., 2012). Furthermore, translation rates decrease dramatically (Kawaguchi et al., 2003, 2004; Branco-Price et al., 2005, 2008; Nicolaï et al., 2006; Mustroph et al., 2009), although often without alteration of specific mRNA levels, which allows a rapid recovery after removal of the stress (Piques et al., 2009; Juntawong and Bailey-Serres, 2012).

These massive alterations on all cellular levels that comprise LES occur during many different stress situations and are therefore discussed as central processes necessary for adaptation. Even though LES involves a collection of phenotypic outcomes, their interconnection and regulation remain to be fully described. Generally, stress adaptation involves both universal and stress specific reactions, indicating that plants perceive multiple stresses and transduces the signal through pathways which may cross-talk at various levels (Chinnusamy et al., 2004). A number of signaling pathways are involved in the regulation of energy utilization and can be linked to the adaptation to stress conditions. Key players of energy signaling are the evolutionary conserved protein kinases sucrose-non-fermenting-1-related protein kinase-1 (SnRK1) and target of rapamycin (TOR). They are proposed to be antagonists in the coordination of energy consumption and preservation (Robaglia et al., 2012) and the balance of their activities might be essential to the regulation of LES in stress adaptation.

## SnRK1 IS A METABOLIC SENSOR KINASE

Sucrose-non-fermenting-1-related protein kinase-1 is a metabolic sensor that can decode energy deficiency signals and induce an extensive metabolic reprogramming. This is mediated by a number of transcription factors and downstream targets that start an energy-saving program at several levels, including transcription, translation or direct phosphorylation of targets (Baena-González et al., 2007). SnRK1 is the plant homolog of the yeast sucrose non-fermenting-1 (SNF1) and the animal AMP-activated protein kinase (AMPK; Halford et al., 2004). SNF1-related protein kinases show close to 50% identity, rising to 65% for the kinase domains (Polge and Thomas, 2007). Their primary role is the integration of nutrient availability, stress signals, and energy expenditure, to be able to activate the required adaptations for homeostasis and survival (Halford and Hardie, 1998; Hardie et al., 1998; Ghillebert et al., 2011). Plants contain two other subfamilies, SnRK2 and SnRK3. They are less similar to SNF1 and AMPK and unique to plants (Halford et al., 2004), and are also involved in plant responses to several stresses (Coello et al., 2011). SnRK2 is involved in ABA signaling, responses to cold, and was shown to improve drought tolerance when overexpressed (Fujita et al., 2009; Halford and Hey, 2009; Yoshida et al., 2014). The SnRK3 family includes *SOS2* (salt overly sensitive 2), involved in conferring salt tolerance (Liu et al., 2000). There is no evidence of redundancy between the different SnRK families and SnRK2 and SnRK3 do not complement the yeast *snf1Δ* deletion mutant growth phenotype (Hrabak et al., 2003). It is clear that they cannot fulfill the role of SnRK1 (Halford and Hey, 2009), even though there is some similarity in target recognition (Zhang et al., 2008). It was suggested that SnRK2 and SnRK3 arose in plants by duplication of SnRK1 and then diverged rapidly during plant evolution to meet new needs related to networks linking stress and ABA signaling with metabolic signaling (Halford and Hey, 2009).

**FIGURE 2:**
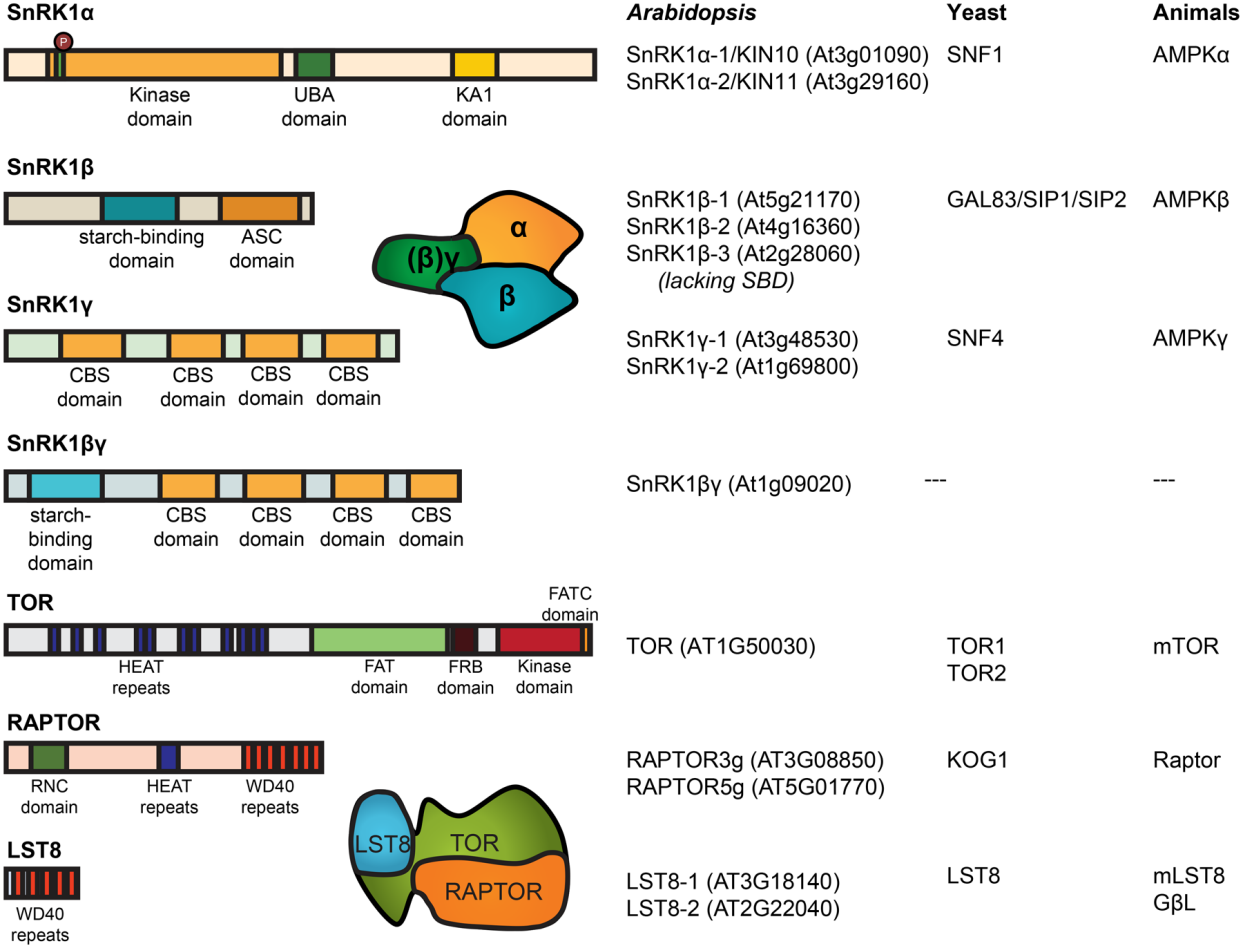
**Domain structure and nomenclature of *Arabidopsis* SnRK1 and TOR subunits.** SnRK1 structures include the conserved phosphorylation sites on T-loop of the α-subunit. The α-subunit contains the kinase domain, together with an auto-inhibitory (UBA) domain and a kinase associated (KA1) domain where the interaction with the ß-subunit takes place. The β-subunit (except in SnRK1ß3) contains a starch-binding domain (Ávila-Castañeda et al., 2014) and binds to the γ-subunit at the association with the SNF1 complex (ASC) domain. The plant-specific βγ-subunit might take the place described for the γ-subunit in mammals and yeast, containing multiple cystathionine β-synthase (CBS) domains. TOR contain two FAT domains (FRAP, ATM, and TRAP) probably constituting the active center, a PI3K kinase domain, a FRB domain (FKB12-rapamycin binding) for interaction with the inhibitor FKB12, and a number of HEAT repeats [huntingtin, elongation factor 3 (EF3), protein phosphatase 2A (PP2A), TOR1] for the interaction with RAPTOR. Next to HEAT repeats, RAPTOR contains a RNC domain (raptor N-terminal conserved/putative caspase domain) and a number of WD40 repeat domains (Hay and Sonenberg, 2004). Nomenclature as described before (Robaglia et al., 2012).

The SnRK1/SNF1/AMPK kinases typically function as heterotrimeric complexes and require a catalytic α-subunit, KIN10 and KIN11 in plants, and regulatory β and γ subunits (**Figure 2**). The non-catalytic subunits are also conserved among the SnRK1/SNF1/AMPK complex. They are likely involved in substrate recognition, subcellular localization, and regulation of the complex activity (Polge and Thomas, 2007; Ghillebert et al., 2011). Interestingly, the *Arabidopsis* AKINβ1, AKINβ2, and AKINβ3 have markedly different expression patterns, which suggests a level of regulation based on interactions targeting the β subunits in response to different signals (Bouly et al., 1999; Gissot et al., 2004).

The activity of SnRK1 depends on phosphorylation in the highly conserved T-loop by upstream kinases (Sugden et al., 1999). In *Arabidopsis*, the protein kinases SnRK1-activating kinase 1 and 2 (AtSnAK1 and AtSnAK2) were shown to complement a yeast triple kinase mutant by restoring SNF1 upstream kinase activity (Shen and Hanley-Bowdoin, 2006). In addition, they phosphorylate non-truncated AtSnRK1 catalytic subunits *in vitro,* making them putative candidates as SnRK1 physiological upstream kinases (Crozet et al., 2010). Even though it is known that SnRK1 activation requires phosphorylation, it has not been clarified how it is affected by the cellular energy level. In contrast to the mammalian AMPK, SnRK1 is not allosterically activated by AMP, but it was shown that T-loop dephosphorylation and the resulting inactivation of the kinase are inhibited by low concentrations of AMP (Sugden et al., 1999). Furthermore, SnRK1 activity is modulated by specific phosphatases. Two clade A type 2C protein phosphatases (PP2C) were recently shown to dephosphorylate and inactivate SnRK1 through interaction with the catalytic subunit (Rodrigues et al., 2013).

The activity of SnRK1 is also inhibited by trehalose-6-phosphate (T6P). The association between trehalose metabolism and sugar-sensing in plants has recently become more evident (Tsai and Gazzarrini, 2014). Despite its role as a carbon source and in stress protection in resurrection plants, fungi, bacteria, and non-vertebrate animals (Elbein et al., 2003; Paul et al., 2008), the amount of trehalose in the majority of plants is too low to perform this function. It was suggested that trehalose has a major role on metabolism, growth, and development, acting as a signal of sugar availability (Schluepmann et al., 2003; Ramon and Rolland, 2007; Gómez et al., 2010). Trehalose is synthesized from UDP-glucose and glucose-6P via the intermediate T6P in a two-step pathway involving trehalose phosphate synthase (TPS) and trehalose phosphate phosphatase (TPP), and degraded by trehalase (Paul et al., 2008). T6P has a distinctive role in metabolic signaling, and class II TPSs, that include AtTPS5-11, are targets of phosphorylation by SnRK1 (Glinski and Weckwerth, 2005). AtTPS5 is induced by sugars and repressed by starvation (Schluepmann et al., 2004), while the opposite is true for AtTPS8-10 (Osuna et al., 2007).

Trehalose-6-phosphate inhibits the catalytic activity of SnRK1 *in vitro* at physiological concentrations, causing expression changes of KIN10 marker genes consistent with an inactivation of SnRK1 (Zhang et al., 2009), while *Arabidopsis* seedlings overexpressing SnRK1 show a glucose-hypersensitive phenotype (Cho et al., 2012), similar to seedlings with low T6P (Schluepmann et al., 2003). However, even though T6P inhibits the growth of *Arabidopsis* seedlings, it does not inhibit SnRK1 catalytic activity in extracts of mature leaves (Zhang et al., 2009), suggesting that an intermediary factor is needed for SnRK1 inhibition by T6P. Recently, it was suggested that T6P and SnRK1 might act through different, but interacting signaling pathways (Lunn et al., 2014) and can play antagonistic roles during stress responses (O’Hara et al., 2013). This is particularly important in stress conditions that negatively affect carbon levels, leading to an activation of starvation responses through SnRK1. For example, T6P levels are much lower in rosettes harvested in the dark and in carbon-starved seedlings (Lunn et al., 2006; Carillo et al., 2013; Yadav et al., 2014). However, stress conditions do not necessarily lead to carbon depletion. For example, under moderate drought or cold stress, a wide range of carbohydrates accumulate in *Arabidopsis* (Muller et al., 2011), including abundant sugars such as hexoses and sucrose, other sugars such as trehalose or mannitol, amino acids, organic acids, structural C-rich compounds like cellulose, among others. In grapevine, sucrose and T6P contents increase in response to chilling (Fernandez et al., 2012). T6P may therefore play a role in the inhibition of SnRK1 under conditions where carbon sources are not limited (Lunn et al., 2014). The cross-talk between SnRK1 and T6P when growth is limited by sink capacity was recently studied by varying temperature and nutrient supply to induce sink limitation, and feed sucrose and glucose at physiological levels (Nunes et al., 2013). In these conditions, T6P responds specifically to sucrose, even at different growth rates. Moreover, there was a strong correlation between T6P- and SnRK1-regulated gene expression, but not between T6P and relative growth rate. It appears that SnRK1 marker gene expression is related to T6P content regardless of the growth outcome, but further investigations will hopefully elucidate the relationship between SnRK1 and T6P.

## SnRK1 REGULATES STRESS RESPONSES UPON LOW ENERGY

The activation of SnRK1 initiates massive transcriptional changes, possibly by affecting a number of transcription factors (Baena-González et al., 2007). The gene expression profile mediated by the SnRK1 subunit KIN10 is positively correlated with the one induced by deprivation of sugar and carbon, and negatively correlated with that controlled by sugars. This places KIN10 as a regulator of gene expression upon starvation and stress conditions. The most prominent KIN10-activated genes represent a variety of major catabolic pathways, including degradation of cell wall, starch, sucrose, amino acid, lipid, and proteins which provide alternative sources of energy and metabolites. Additionally, a large set of genes involved in energy-consuming ribosome biogenesis and anabolism are repressed.

**FIGURE 3:**
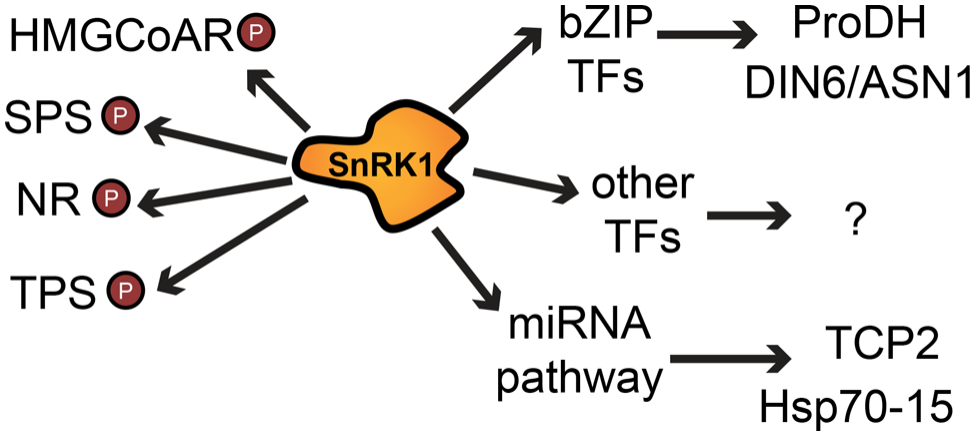
**Simplified summary of SnRK1 effects on cellular processes by direct phosphorylation of target proteins and by alteration of mRNA levels of many genes via transcription factors or the miRNA machinery.** Arrows indicate a positive or negative regulatory effect; P denotes phosphorylation. Abbreviations: HMGCoAR, 3-hydroxy-3- methylglutaryl-coenzyme A reductase; SPS, sucrose phosphate synthase; NR, nitrate reductase; TPS, trehalose phosphate synthase; bZIP TF, basic leucine zipper transcription factor; ProDH, proline dehydrogenase; DIN6/ASN1, dark inducible 6/asparagine synthetase 1; TCP2, teosinte branched 1, cycloidea and PCF transcription factor 2; Hsp70–15, heat shock protein 70–15.

From the possible mechanisms by which SnRK1 affects transcription, the members of the S1 class of the basic leucine zipper (bZIP) transcription factors are probably the best described (**Figure 3**). They belong to a large family of several classes in eukaryotes (Reinke et al., 2013) and function as homo-or heterodimers, which increases their potential for regulation (Jakoby et al., 2002; Corrêa et al., 2008; Schütze et al., 2008). From the bZIP family, bZIP1, bZIP11, and bZIP53 were proposed to mediate some of the transcriptional changes induced by the SnRK1 signaling pathway (Baena-González et al., 2007) and could be linked to the regulation of LES. In the presence of sucrose and glucose, the transcript levels of bZIP1 and bZIP53 decrease, and energy availability also seems to affect the phenotypes of their mutants. bZIP53 overexpression results in reduced plant size, delayed bolting and expression of seed-specific genes in leaves (Alonso et al., 2009). bZIP1 knockout plants were shown to grow faster than wild type on medium lacking glucose (Kang et al., 2010), while a plant overexpressing bZIP1 showed a stronger starvation response indicated by faster leaf-yellowing in extended night conditions (Dietrich et al., 2011). Under ambient growth conditions, bZIP1 gene expression is limited to sink tissue like pollen or young leaves (Weltmeier et al., 2009), but transcript levels were shown to be increased in source leaves after sugar starvation induced by extended night (Dietrich et al., 2011), while bZIP11 transcript levels increase in the presence of glucose and sucrose (Rook et al., 1998). On the other hand, bZIP11 translation is repressed by sucrose mediated by an upstream open reading frame (Wiese et al., 2004; Rahmani et al., 2009). This decrease is observed at physiological sucrose levels in most tissues, restricting bZIP11 activity to conditions of low energy availability (Wiese et al., 2004). Transgenic plants overexpressing bZIP11 show reduced plant size, seed production, viability and a wide effect on gene regulation and metabolism as demonstrated by microarray analysis of these plants (Hanson et al., 2008).

Several gene expression studies identified putative target genes of these transcription factors and there is considerable overlap between their targets and genes regulated by SnRK1. bZIP11 targets include several genes associated with LES, involved in the regulation of trehalose and other minor regulatory carbohydrates, such as myo-inositol and raffinose (Ma et al., 2011). bZIP11 also induces *GDH1* and *GDH2*, genes encoding glutamate dehydrogenase (Hanson et al., 2008). The double mutant *gdh1gdh2* was shown to be more susceptible to extended night, likely due to the role of these enzymes in amino acid degradation (Miyashita and Good, 2008a,b). Other genes involved in amino acid metabolism were shown to be regulated by bZIP transcription factors. Members of the S1 class specifically activate the G-box containing promoter of the SnRK1 regulated gene *DIN6/ASN1* (Baena-González et al., 2007). They were also shown to activate gene expression of proline dehydrogenase (ProDH; Satoh et al., 2004) by binding to G-boxes contained in the promoter region (Weltmeier et al., 2006; Dietrich et al., 2011). While ProDH is thought to be involved in stress recovery (Weltmeier et al., 2006), the accumulation of asparagine and other amino acids during dark-induced starvation was proposed to result from protein degradation in order to provide an alternative to carbon as energy source (Caldana et al., 2011; Dietrich et al., 2011).

Basic leucine zipper transcription factors mediate many, but not all SnRK1 effects on transcription. It remains to be studied which factors mediate the first, direct effect on gene expression and which genes are regulated by secondary mechanisms. Further studies will hopefully reveal a number of additional transcription factors involved in SnRK1 regulation of gene expression.

Sucrose-non-fermenting-1-related protein kinase-1 also affects enzymes by direct phosphorylation. For example, it inhibits the activity of HMG-CoA reductase, the rate-limiting step in sterol synthesis (Clarke and Hardie, 1990; Mackintosh et al., 1992). Two other enzymes were shown to be substrates of SnRK1, sucrose phosphate synthase (SPS), and nitrate reductase (NR; McMichael et al., 1995; Douglas et al., 1997) which are key biosynthetic enzymes involved in the control of nitrogen assimilation and sucrose synthesis (**Figure 3**).

In addition to transcriptional changes and direct phosphorylation, SnRK1 was recently shown to activate a miRNA pathway (Confraria et al., 2013). Some of the candidate miRNA targets can be connected to the SnRK1 pathway and miRNAs can therefore be placed as components of the SnRK1 signaling pathway, as they regulate mRNA targets and possibly tune down specific cellular processes during the stress response (**Figure 3**). Most of the affected genes correspond to genes related to ribosomal proteins (RPs) and translation, which is in accordance with the role of SnRK1 as a repressor of biosynthetic processes and as a modulator of energy metabolism (Baena-González et al., 2007; Baena-González and Sheen, 2008). In animals there seems to be a link between miRNAs and metabolism: AMPK activation was recently reported to induce the differential accumulation of multiple miRNAs (Liu et al., 2013a), suggesting that miRNAs could be possible common elements in diverse organisms for restoring homeostasis following stress (Confraria et al., 2013). A recent paper supports this view by showing a strong connection between the regulation of miRNA expression and glucose-mediated regulatory responses (Duarte et al., 2013). These recent findings make it clear that SnRK1 mode of action goes beyond direct phosphorylation or modulation of transcription and promise new discoveries to come on the interplay between multiple pathways in the regulation of the LES.

## THE TOR KINASE IS INVOLVED IN LES

Another key component in this network is the serine/threonine kinase TOR. TOR kinase genes are present in every eukaryote genome analyzed so far and they share 40–60% sequence identity (De Virgilio and Loewith, 2006; Wullschleger et al., 2006). These large proteins are well described for their central roles in the energy signaling pathways of yeast, mammals and plants (Wullschleger et al., 2006; Robaglia et al., 2012). TOR is activated, in both yeast and mammals, by high amino acid levels, but inactivated under amino acid starvation (Jewell et al., 2013). In plants, TOR activity has been linked to cell and organ size, seed yield, and stress resistance (Ren et al., 2012) and it was suggested to play a role in the regulation of carbon partitioning and growth (Zhang et al., 2013). Furthermore, it has been shown that TOR and SnRK1 interact closely and act in opposite ways in the regulation of nutrient-driven processes like autophagy (Robaglia et al., 2012).

In yeast and mammals, TOR functions in two complexes with distinct functions, TOR complex 1 (TORC1) and TORC2, characterized by different interaction partners. Of these, only subunits of TORC1 (TOR, LST8/GbetaL and KOG1/RAPTOR) are present in the *Arabidopsis* genome (**Figure 2**; van Dam et al., 2011). Mutations that disrupt TOR or RAPTOR genes were shown to be embryo lethal (Menand et al., 2002; Deprost et al., 2005). In addition, the low sensitivity of the *Arabidopsis* TOR toward rapamycin (Xiong and Sheen, 2012; Caldana et al., 2013) makes the study of TOR signaling in plants more difficult than in other organisms, where the inhibitor was extensively used to study TOR functions (Wullschleger et al., 2006). Alternative approaches were developed including the expression of a yeast FKBP12 protein which confers rapamycin sensitivity (Sormani et al., 2007; Ren et al., 2012), modulation of TOR expression by overexpressor or RNA interference constructs (Deprost et al., 2007; Ren et al., 2011) and the expression of an inducible artificial microRNA targeting TOR (Caldana et al., 2013).

The *Arabidopsis* TOR promoter is active in root and apical primary meristems, embryo and endosperm, but not in source leaves or differentiated cells (Menand et al., 2002). TOR acts on cell cycle control in *Arabidopsis* root meristems by directly phosphorylating the transcription factor E2Fa that regulates S-phase gene expression (Xiong et al., 2013). It was proposed as a potential integrator of cell cycle, cell expansion and cytoplasmic growth (Sablowski and Carnier Dornelas, 2014) and could thus be responsible for the activation of growth in the meristems in response to sugars provided by the photosynthetic source tissues (Xiong et al., 2013). Furthermore, TOR inhibition leads to a reduction of the length of the root meristematic zone and the division zone therein (Montané and Menand, 2013). Recently, TOR was shown to be activated by the growth hormone auxin and is involved in the regulation of translation of auxin responsive genes (Bögre et al., 2013; Schepetilnikov et al., 2013). Accordingly, reduction of TOR expression in *Arabidopsis* results in severe growth arrest with plants displaying decreased cell size, whereas TOR overexpression leads to an increase in shoot and root growth (Deprost et al., 2007).

TOR complex 1 was described to be involved in the control of transcription, protein synthesis, and autophagy in yeast and mammals (Martin and Hall, 2005; De Virgilio and Loewith, 2006; Wang and Proud, 2011). In *Arabidopsis*, massive transcriptional changes induced by glucose in seedlings are dependent on TOR signaling. The genes activated by this signaling pathway are involved in amino acid synthesis, translation, glycolysis and the TCA cycle, cell wall synthesis and modification, whereas genes involved in protein and amino acid degradation and autophagy regulation were downregulated (Xiong et al., 2013). The role of TOR signaling in the induction of biosynthesis and the repression of catabolic pathways was underlined by RNA sequencing and microarray analysis studying gene expression changes in response to TOR inactivation (Ren et al., 2012; Caldana et al., 2013). These transcriptional changes were shown to be accompanied by an increase in starch content (Moreau et al., 2012) and an accumulation of organic and amino acids (Ren et al., 2012; Caldana et al., 2013), as well as a decrease in galactinol and raffinose levels (Moreau et al., 2012). This led to the conclusion that TOR downregulation mimics starvation (Caldana et al., 2013) and strengthens the importance of the TOR pathway in starvation responses.

Furthermore, the TOR kinase was implicated in sugar signaling pathways to control translation. Protein synthesis is a very energy demanding process and therefore needs to be tightly regulated in function of the cellular energy availability. In mammals and yeast, TORC1 affects the level of rRNA (Claypool et al., 2004; Mayer et al., 2004; Li et al., 2006a; Tsang et al., 2010) and RP gene expression (Jorgensen et al., 2004; Martin et al., 2004; Rudra and Warner, 2004), as well as cap-dependent translation (Ma and Blenis, 2009) and scanning along structured 5′-UTRs (Meyuhas and Dreazen, 2009).

The tight regulation of translation according to energy availability also occurs in plants (Pal et al., 2013; Lastdrager et al., 2014). For some of the well described pathway components, like the phosphorylation of 4E-BP, evidence in plants is lacking (Van Der Kelen et al., 2009; Ren et al., 2011). Nevertheless, plants expressing RNAi constructs for TOR or its positive downstream effector TAP46 displayed a significant decrease in polysomal loading and in protein synthesis (Ahn et al., 2011). The transcription of RP genes is induced after sugar treatment and seems to be dependent on the diurnal cycle (Bläsing et al., 2005; Usadel et al., 2008; Baerenfaller et al., 2012) and glucose induction of RP gene expression was shown to depend on TOR activity (Xiong et al., 2013). Furthermore, TOR overexpression was shown to induce rRNA production and ChIP experiments showed that TOR binds directly to the 45S promoter region (Ren et al., 2011). TOR mediated ribosomal protein S6 kinase (S6K) activity was proposed to be important to maintain eukaryotic translation initiation factor 3 subunit H (eIF3h) phosphorylation, which is needed for translation reinitiation and thus for translation of uORF containing mRNAs (Schepetilnikov et al., 2013). Starvation inhibits the TOR kinase and therefore allows energy costly ribosome biogenesis and translation processes to be reduced in growth limiting conditions (Ma and Blenis, 2009; Robaglia et al., 2012).

## INTERACTION BETWEEN SnRK1 AND TOR SIGNALING

**FIGURE 4:**
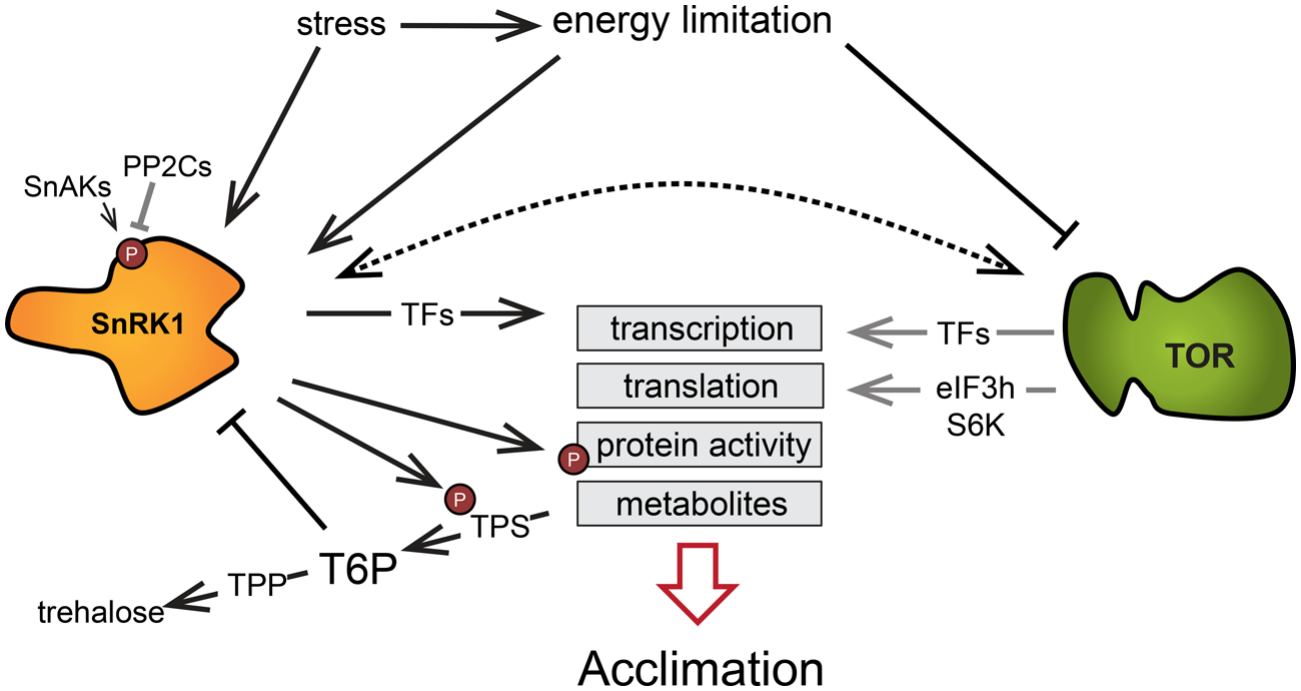
**The low energy syndrome involves processes regulated by TOR and SnRK1 central to the adaptation to energy limitation.** The kinases SnRK1 and TOR are affected by energy availability and mediate transcription, translation, enzyme activity, and the accumulation of metabolites in order to drive adaptation to different conditions. Gray arrows indicate processes shut down in response to energy limitation and the dashed arrow denotes a probable interaction between these two signaling pathways. Abbreviations: SnAK, SnRK1-activating kinase; PP2C, type 2C protein phosphatase; TPS, trehalose phosphate synthase; T6P, trehalose 6-phosphate; TPP, trehalose phosphate phosphatase; eIF3h, eukaryotic translation initiation factor 3 subunit H; S6K, ribosomal protein S6 kinase.

The TOR and SnRK1 signaling pathways have emerged as crucial in regulating the perception and responses to nutrient and energy levels. The TOR kinase is activated in favorable nutritional and energy conditions, while SnRK1 is stimulated upon nutrient and energy starvation. It is becoming increasingly clear that they act in opposite ways in the regulation of nutrient-driven processes, such as autophagy. For example, SnRK1 induces autophagy to promote recycling of cytosolic components in response to situations where C or N metabolites are in short supply. Conversely, TOR restrains autophagy in energy-replete conditions and is involved in the regulation of N assimilation and in the synthesis of C metabolites like starch or raffinose (Robaglia et al., 2012). They both regulate many similar processes in the context of LES (**Figure 4**) and massively affect the transcription of a number of genes. By comparing the expression of genes targeted by SnRK1/KIN10 (Baena-González et al., 2007) and TOR (Xiong et al., 2013) in two available transcriptional datasets (data was acquired from protoplast and seedlings, respectively), we found that there was a significant overlap in the genes affected by both TOR and SnRK1. More than half of the genes (294 out of 507) described as KIN10 upregulated target genes were found to be downregulated by glucose in a TOR-dependent manner. Interestingly, 47 genes which were oppositely affected by TOR and SnRK1 are annotated to encode RPs or proteins related to translation. Furthermore, a similar proportion of genes found to be downregulated by KIN10 (260 of 515 genes) were among the putative upregulated targets of the TOR kinase, including genes annotated to be involved in amino acid metabolism and involved in carbohydrate metabolism. This underlines the hypothesis that TOR and SnRK1 act antagonistically in the regulation of central processes such as translation and carbohydrate and amino acid metabolism, a role that is likely to be crucial during the establishment of stress responses.

Target of rapamycin and SnRK1 are both evolutionary conserved and have been shown to interact in mammalian systems. AMPK is described to regulate the mTOR complex 1 in different ways, by phosphorylating components of the mTOR signaling pathway (Xu et al., 2012). The phosphorylation of the TSC2/TSC1 complex by AMPK leads to the inactivation of Rheb, a GTPase that activates mTORC1. Furthermore, AMPK has been described to directly regulate the Raptor subunit of the mTORC1 complex (Inoki et al., 2012). mTOR and AMPK have opposite roles in the regulation of autophagy by targeting different phosphorylation sites of ULK1 (Kim et al., 2011). Under nutrient limitation, AMPK activates ULK1 and autophagy, whereas mTORC1 inactivates ULK1 in nutrient-rich conditions (Inoki et al., 2012). In plants, some of the described factors are present, including the *Arabidopsis* TCTP that was proposed as a regulator of Rheb in TOR signaling (Berkowitz et al., 2008) and the AMPK phosphorylation site in Raptor (Robaglia et al., 2012). However, their role in the interaction between the TOR and SnRK1 signaling pathways remains to be further analyzed. Additionally, many of the described genes are missing in *Arabidopsis*, such as Rheb itself, TSC1, and TSC2 (van Dam et al., 2011). Their functions were taken over by other factors, indicating that plants evolved energy signaling pathways different from mammals or yeast that remain to be fully described (Xiong and Sheen, 2014).

## TOWARD A BETTER UNDERSTANDING OF LES

The studies discussed above underline the central role of the energy signaling network composed of TOR, SnRK1, bZIP transcription factors, and T6P in the control of growth and development. Exposure of plants to conditions that challenge their energy homeostasis results in significant metabolic reprogramming to prevent damage to cells, tissue, and organs. LES involves various changes in transcription, translation, enzymatic activities, and metabolite levels ultimately aiming at the adaptation to energy deprivation (**Figure 4**). Amongst others, this leads to protein, lipid, and chlorophyll breakdown (Contento et al., 2004; Thimm et al., 2004), while in parallel, carbon utilization is inhibited, which, taken together, severely affects growth (Gibon et al., 2004).

Even though much is already known about the LES network, there are still many open questions. More experimental data is needed to unravel unknown mechanisms behind LES, but several experimental limitations may be restraining new discoveries. For example, most studies were conducted in protoplasts, whole seedlings or plant tissue without distinguishing source and sink organs. Since the interplay of molecules is complex and coordinated in both time and space, increasing spatial resolution of metabolites with the use of better techniques, like subcellular fractionation (Nägele and Heyer, 2013), is necessary to enable a better understanding of regulation of stress conditions by allowing tissue-, cell-, and compartment specific analyses of metabolic changes. Furthermore, the pleiotropic nature of SnRK1 and TOR limits the use of mutants as mutations result in undesired effects that can hardly be precluded. The use of constitutive promoters for overexpression leads to the loss of information about the localization of the expression of a certain gene. This information is very important as many of the discussed genes are differentially expressed between source or sink tissues, which likely impacts on their role and function. It would thus be useful to develop additional mutants, especially including organ and temporal specific promoters, to overcome these current issues. The use of inducible systems is of high interest for both SnRK1 and TOR, but particularly for the latter since knock-out mutants are not available due to lethality. Hence, the uses of inducible artificial microRNAs are a valuable option to down-regulate its expression.

The study of networks like LES requires the analysis of complex datasets. It is important to realize that the challenge is not only the acquisition of more and better experimental data, but especially to be able to integrate it to facilitate its interpretation via methods often referred to as systems biology or omics-based approaches. To improve upon current approaches, theoretical methods of uni- and multivariate statistics and mathematical modeling have been developed which now allow large scale analysis of biological networks (Nägele and Weckwerth, 2012). Several problems related to plant science are being addressed using these approaches, including responses to stress, (Yamaguchi-Shinozaki and Shinozaki, 2006), plant defense (Li et al., 2006b) and the identification of new players, such as transcription factors (Hirai et al., 2007).

Low energy signaling is a complex network that integrates multiple cellular and environmental signals and comprises several cellular changes. To avoid limitation in the interpretation of the biological events, it is important to study this pathway on all possible levels, comprising the gene, transcript, protein, and metabolite levels. Limitations can arise, for example, from the consideration of only transcriptomic data. With this type of data, differences in transcription and RNA stability cannot be distinguished. Also, changes in transcript levels often do not reflect changes in protein or metabolic levels and little information is obtained at this level. Substantial contributions have been made mainly concerning the integration of transcript and metabolite data for *A. thaliana* (Hirai et al., 2004, 2005; Tohge et al., 2005). By combining data from different molecular levels, a better understanding of pathway regulation being affected under low energy conditions and contributing to the acclimatory response will be significantly promoted. One type of systems biology approach is based on the collection of data from different platforms followed by data driven integration using advanced statistical models to study the dynamic interactions between components (Yuan et al., 2008; Fukushima et al., 2009; Keurentjes, 2009). Another more targeted approach where a specific regulatory model involving all known molecular players is built uses mathematical modeling to advance understanding of biological processes (Pokhilko et al., 2010, 2012, 2013; Gould et al., 2013; Seaton et al., 2014). The interaction between predictive models and experimental confirmation can be very effective, as having a more directed approach in a given experiment could enable the achievement of faster and more targeted results. Furthermore, unknown components of the studied system can be discovered (Dalchau et al., 2011). Some of the components that comprise the LES network may still be unknown, but this type of approach could provide very useful information especially in the connection between TOR and SnRK1.

The use of systems biology is limited by the availability of data and requires generalization, simplification, and assumptions. However, it clearly has great potential to increasingly contribute to the understanding of biological networks, including the LES pathway, in combination with other approaches that involve cell biology, biochemistry, or genetics. TOR and SnRK1 are related in the processes they regulate and are activated under opposite conditions, but so far there is no indication in plants that they or their targets interact directly to optimize the activation and repression of certain processes. While in mammals AMPK phosphorylates the TSC2/TSC1 complex and Raptor, TSC2 and the AMPK phosphorylation site in Raptor are missing in *Arabidopsis* and it is not yet clear whether TOR and SnRK1 pathways interact in a similar way. It is possible that despite their connection, TOR down-regulation is just another way to limit energy use, independent from SnRK1. However, given the complexity of the network and the interconnections seen so far, not only in plants but also in other systems, it seems more likely that additional links or factors are yet to be unraveled. It is crucial to establish if there is a direct interaction between TOR and SnRK1, or if they act independently. In this context, a better understanding of their (sub-)cellular localization could provide insights to their mode of action and possible interaction. Protein interaction studies may uncover novel interactions between known LES components or even unravel new components of the network. Their role in the metabolic reprogramming induced by energy deprivation may then be tested by metabolomics studies which directly give information about changes in the concentrations of central metabolite pools. A multidisciplinary approach on various levels of cellular and organismal organization is needed to be able to draw a comprehensive picture about low energy induced metabolic reprogramming.

## AUTHOR CONTRIBUTIONS

Filipa Tomé is first author, Thomas Nägele is second author, Magdalena Gamm is last author, and all other authors contributed equally to the work.

## Conflict of Interest Statement

The authors declare that the research was conducted in the absence of any commercial or financial relationships that could be construed as a potential conflict of interest.
